# A Summative Usability Evaluation of an Infusion Pump Through Simulation-Based Testing With Nurses: Mixed Approach Study

**DOI:** 10.2196/86443

**Published:** 2026-03-09

**Authors:** Yingjun Wan, Yun Zhang, Yunlong Zhao, Han Yu, Kaifeng Liu

**Affiliations:** 1 Academy of Medical Engineering and Translational Medicine Tianjin University Tianjin China; 2 State Key Laboratory of Advanced Medical Materials and Devices Tianjin University Tianjin, Tianjin China; 3 Tianjin Key Laboratory of Quality Control and Evaluation Technology for Medical Devices Tianjin China

**Keywords:** usability, medical device, infusion pump, simulated use, use error

## Abstract

**Background:**

Suboptimal design of infusion pumps may lead to usage errors, thereby compromising patient safety. Usability evaluation enables medical device design teams to identify and rectify design-related usability issues in a timely manner. Nevertheless, existing research on infusion pump usability continues to exhibit limitations in aspects such as task design.

**Objective:**

The study aimed to evaluate the usability of an infusion pump (SLGO SP-200 [SLGO Medical Technology Co, Ltd]) through simulation-based testing with nurses in a usability laboratory designed to simulate an intensive care unit.

**Methods:**

A total of 12 registered nurses with experience in using infusion pumps participated in this study. Nurses were asked to perform 12 operational tasks using the infusion pump. The participants were also asked to perform 7 knowledge tasks, where they were required to find relevant information in the user manual. Participants’ behavioral measures (task completion time, frequency of manual query, frequency of asking for assistance from researchers, frequencies of operation difficulties, near-misses, and failures), perceptions (perceived ease of use, perceived concentration level required, perceived likelihood of making programming errors, perceived mental workload, satisfaction, and use intention) were collected to evaluate the usability and identify interface design deficiencies of the pump.

**Results:**

The study found that the participants were generally able to complete the tasks. All operational tasks were completed within 3 minutes, and all knowledge tasks were completed within 2 minutes. Our study identified 79 difficult operations, 9 near-miss operations, and 36 operation failures. The causes of the above problems were analyzed. Participants generally found the infusion pump to be user-friendly, requiring a medium level of attention resources, and reported low levels of mental workload and likelihood of making programming errors.

**Conclusions:**

The study results can provide a basis for the design of infusion pumps, help practitioners define the risks of use and the key content of training, and provide an important reference for the design of usability evaluation schemes for medical devices.

## Introduction

Infusion pumps are medical devices that deliver fluids, medications, or nutrients directly into the patient body in a controlled manner [1]. Well-designed infusion pumps of good quality are necessary for providing effective and safe patient care. However, infusion pumps are often involved in incidents and accidents [2-4]. A White Paper published by the US Food and Drug Administration (FDA) in 2010 (White Paper: Infusion Pump Improvement Initiative) indicated that between 2005 and 2009, the FDA received approximately 56,000 adverse event reports related to the use of infusion pumps, and there were 87 infusion pump models recalled due to safety concerns [4]. Other studies have indicated that the number of adverse events or near-miss incidents occurring during the use of medical devices is significantly higher than the data reported officially [5-7].

The poor human-machine interface design of medical devices (eg, unclear information presentation and ambiguous alarms) is one of the main reasons for use errors [5,8-11]. To address this issue, development teams for medical devices like infusion pumps need to incorporate usability engineering, which focuses on safety, efficiency, and user satisfaction during the use of medical devices [12,13]. Medical device regulators worldwide have published various guidelines and standards to integrate usability engineering into the device approval process [14]. In 2001, the American National Standards Institute (ANSI) and the Association for the Advancement of Medical Instrumentation (AAMI) released ANSI/AAMI HE74:2001, which described the usability engineering design process for medical devices; it was one of the earliest standards emphasizing usability [15]. In 2007, the International Electrotechnical Commission (IEC) released IEC 62366, detailing the usability engineering design process for medical devices [16]. This standard was updated in 2015 to further elaborate on the design process, considering risk management. In China, usability engineering for medical devices is still in its early stages. In 2020, the Center for Medical Device Evaluation of the National Medical Products Administration organized the drafting of the guidelines for technical review of human factors design for medical devices (draft for comments). In March 2024, the guideline was officially published, providing standards and regulations that define how usability testing should take place [17].

Usability testing is a crucial process within usability engineering, which consists of formative and summative testing. Formative testing is primarily conducted during the design and development phases of medical devices, while summative testing is conducted after the development phase to ensure that the designed device sufficiently meets user needs [18]. Simulated use testing is a common method of usability testing, where representative users are invited to use the medical device to perform representative tasks in a realistic setting [19-21]. This method allows for the observation and collection of user behavior data, revealing interface design flaws and identifying interaction strengths and opportunities for improvement.

Several previous studies have empirically evaluated the usability of infusion pumps [5,21-23]. For instance, Garmer et al [5] conducted a usability experiment to compare 2 interfaces for an infusion pump. Experimental tasks included setting or adjusting a flow rate or volume to be infused and starting or stopping an infusion. Liu et al [21] performed a heuristic evaluation and simulated use testing to compare the usability level of 4 infusion pumps to inform pump selection. Trbovich et al [22] examined how traditional pump, smart pump, and smart pump with barcode affected nurses’ ability to safely administer intravenous medications. Schmettow et al [23] conducted a usability study to compare a novel syringe infusion pump prototype to an existing design. However, existing research still faces a series of limitations. First, previous usability evaluations only considered operational tasks related to pump use, while neglecting knowledge tasks associated with the use of the instruction manual. As an integral component of the system, the clarity, comprehensibility, accessibility, and instructional value of the manual also require evaluation [24]. Second, most evaluation tasks focused solely on core functions, such as infusion programming, with insufficient attention given to alarm handling and accessories installation. Third, current evaluations focused on observing operational difficulties without summarizing the underlying causes and potential risks of these issues. Through linking identified usability issues to potential use-related risks, it can offer a direct pathway from evaluation findings to specific, actionable design improvements aimed at risk mitigation [23].

The importance of usability testing for medical devices is gaining attention among professionals worldwide, highlighting the urgent need for research to establish guidelines for typical usability testing methods, processes, and data analysis. In light of this, this study invited registered nurses to conduct a summative usability test on a new infusion pump, aiming to identify human-machine interface design flaws and provide guidance for the design, evaluation, training, and use of infusion pumps.

## Methods

### Design

**Figure 1:**
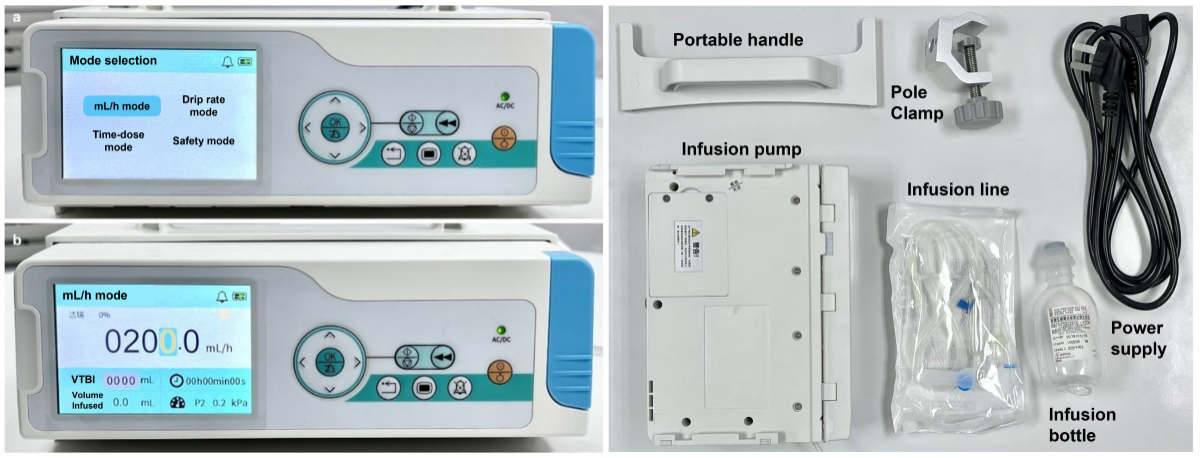
The infusion pump being evaluated—main interface (left) and its accessories (right).

A summative usability evaluation of an infusion pump (SLGO SP-200, SLGO Medical Technology Co, Ltd; Figure 1) was conducted, where participants were required to complete a series of operational tasks and knowledge tasks in a simulated intensive care unit (ICU). During the evaluation, behavioral data (eg, task completion time, frequency of asking for help, frequency of difficult operation, frequency of near-miss operation, and frequency of operation failure) as well as subjective data (eg, subjective perceptions and interviews) were collected.

### Participants

A total of 12 nurses with experience in using infusion pumps were recruited from 2 hospitals in Tianjin, China. The participants were stratified by clinical unit (specialized or general hospitals), department (ICU, operating room, or medicine), and clinical experience (≤8 y or >8 y) [21]. The participants were aged between 25 and 55 years (mean age 35.3, SD 8.1 y). Previous literature in usability engineering indicated that 80%-90% of usability problems can be detected with 5-8 participants; beyond this point, the incremental yield of new issues diminishes significantly [25,26]. All participants received training on the infusion pump being evaluated before the evaluation.

### Environment and Apparatus

**Figure 2:**
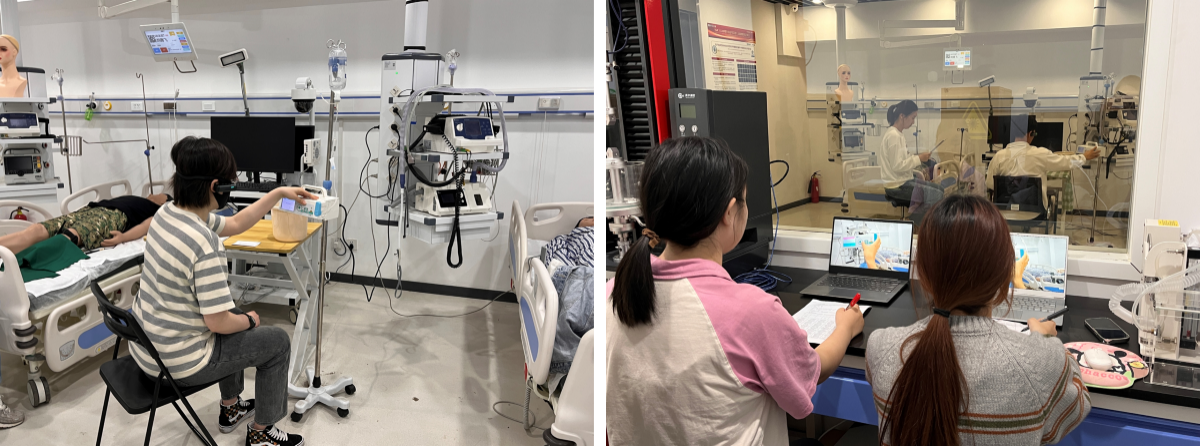
The testing room (left) and the observation room (right) of the usability laboratory.

The experiment was conducted in a usability laboratory designed to simulate an ICU. The laboratory consists of a testing room and an observation room, separated by a one-way glass. Participants performed experimental tasks in the testing room while wearing a head-mounted camera (Lenovo-Lx918) to continuously record their operations in real time. Observers in the observation room monitored the participant’s operations through a computer screen displaying the live feed from the head-mounted camera (Figure 2).

### Task

A total of 12 operational tasks were designed (Table 1), including accessories installation, infusion programming, system configuration, and alarm handling. The 2 alarm handling tasks (tasks 8 and 10) were predesigned as part of the scenario, and the alarms were manually triggered by a research assistant at a planned point during the participant posttask interview. This approach allowed researchers to integrate the alarm response naturally into the ongoing tasks without relying on the participant to inadvertently cause the alert condition. Specifically, the air bubble alarm (task 8) was triggered by the research assistant intentionally introducing a small air bubble into the infusion line. No power connection alarm (task 10) was triggered by the research assistant disconnecting the pump from its power source. Participants were not informed in advance that these alarms would occur, so that we could assess their natural recognition and response to unexpected yet clinically relevant alarms. Additionally, we designed 7 knowledge tasks (Table 2), where participants were required to find relevant information in the user manual.

**Table 1:** The 12 operational tasks.

Task	Task description	Standard operation steps
1	Please install the pole clamp and portable handle on the infusion pump. Then fix the pump securely on the pole stand.	Install the pole clamp Install the portable handle Fix the pump on the pole stand
2	Please install the power supply and turn on the infusion pump.	Install the power supply Turn on the pump
3	Please install the infusion line.	Push the pump door latch outward and fold down the front panel Install the infusion line horizontally from right to left and secure it into the guide port Close the pump door
4	Please adjust the brightness level of the screen to the highest level.	Press the menu key to enter the system setup interface Select the key for brightness settings Use the directional keys to adjust the level
5	Please open the roller clamp on the infusion line. Set the drip rate to 50 drops/min in drip mode. Start infusion after priming.	Open the roller clamp Select the drip rate mode on the main interface Set the drip rate to 50 drops/min Press the prime and bolus key to purge air from the infusion line Press the start and stop key to start the infusion
6	Please set the volume to be infused to 58 mL and the infusion duration to 60 min in the time-dose mode, then start the infusion.	Press the start and stop key to pause the previous infusion Press the return key to enter to the main interface Select the time-dose mode Set the volume to 58 mL and the duration to 60 min Press the start and stop key to start the infusion
7	Please adjust the flow rate to 254.5 mL/h in the mL/h mode and then start the infusion.	Press the start and stop key to pause the previous infusion Press the return key to enter to the main interface Select the mL/h mode Set the flow rate to 254.5 mL/h Press the start and stop key to start the infusion
8	Please address the current alarm (ie, air bubble alarm) and then continue the infusion.	Press the return key Press the prime and bolus key until bubbles are discharged from the very end of the infusion line. Press the start and stop key to restart the infusion.
9	Please set the flow rate to 99 mL/h and the preset volume to 1 mL in safe mode, then start the infusion.	Press the start and stop key to pause the previous infusion. Press the return key to enter to the main interface. Select the safe mode. Set the flow rate to 99 mL/h and preset volume to 1 mL Press the start and stop key to start the infusion
10	Please address the current alarm (no power connection).	Press the start and stop key to pause the previous infusion Connect the power supply to the pump
11	Please turn off the pump, remove the infusion line, disconnect the power, and take the infusion pump off the stand.	Turn off the pump Disconnect the power supply, first the AC power, then the connection to the infusion pump Remove the infusion line and close the pump door Take the infusion pump off the stand
12	Please remove the pole clamp and the portable handle from the infusion pump.	Remove the pole clamp Remove the portable handle

**Table 2:** The 7 knowledge tasks.

Task	Question	Answer
1	What is the minimum distance required between the infusion pump and high-frequency surgical equipment?	At least 0.5 m
2	What are the contraindications for the use of the infusion pump?	It is prohibited for blood transfusion, insulin infusion, as well as analgesia, chemotherapy, and epidural anesthesia infusion
3	What are the infusion pump’s alarm levels and their corresponding indicator colors?	Red for high priority, yellow for low priority
4	What is the normal operating temperature range for the infusion pump?	+5 °C to +40 °C
5	What is the required distance range between the infusion bottle and the infusion pump?	30 cm to 50 cm
6	Please confirm the meaning of the symbol. 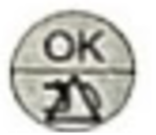	Confirm and alarm reset key. In the parameter setting interface, it cycles through editable parameters; during an alarm, it performs an alarm reset operation.
7	What is the troubleshooting method for an “Occlusion” alarm on the infusion pump?	Press the start and stop key to pause the infusion. Check if the tubing is kinked, if the roller clamp is closed, or if the needle is blocked. If the issue is due to medication viscosity, the pressure setting can be adjusted.

### Procedures

Upon arriving at the laboratory, the researcher first introduced the objective and procedure of the evaluation to the participants. Participants were required to complete 12 operational tasks and verbally report “task completed” after finishing each one. They were instructed to use the infusion pump in the same manner as they would in their daily work. If they encountered difficulties, they could refer to the manual. If they were still unable to solve the problem after referring to the manual, they could seek assistance from the researchers. Participants were also required to complete 7 knowledge tasks, in which they were required to search the manual for relevant information and answer the questions. After completion of all tasks, participants filled out a questionnaire [21,27], which included 6 dimensions—perceived ease of use, perceived mental workload, perceived concentration level required, perceived likelihood of making programming errors, satisfaction, and use intention (Table 3). All items were measured using a 7-point Likert scale (1=strongly disagree and 7=strongly agree). Posttask interviews were held to gather overall user experience and suggestions for pump design. For operational tasks, interview questions included “Do you recall any errors you made while performing the tasks?,” “Can you remember any instances where you almost made a mistake but realized it immediately?,” “What is your overall impression of using this infusion pump for the first time?,” and “Do you have any suggestions for improving this infusion pump?” For knowledge tasks, interview questions included “Did you find it easy to locate the relevant information?,” “How would you evaluate the clarity of the corresponding content in the user manual?,” and “Do you have any suggestions for improving the content of the user manual?” The duration of the experiment was approximately 60 minutes.

**Table 3:** Items used to measure participants’ perceptions.

Dimensions	Items
Perceived ease of use	Item 1: It is easy to prime and load a set. Item 2: It is easy to load and unload a set. Item 3: It is easy to program a basic infusion. Item 4: It is easy to program a drug infusion from scratch. Item 5: It is easy to titrate ﬂow rates. Item 6: It is easy to turn the pump on and oﬀ.
Perceived mental workload	Item 7: Setting up and programming the infusion pump require a high level of mental workload.
Perceived concentration required	Item 8: Using this infusion pump requires my high level of concentration.
Perceived likelihood of making programming errors	Item 9: The design of this infusion pump is complex, making it easy for me to make errors.
Satisfaction	Item 10: Overall, I am satisfied with this infusion pump.
Use intention	Item 11: Overall, I am willing to use this infusion pump.

### Data Analysis

Data on task completion time, frequency of manual query, frequency of asking for assistance, and frequencies of difficult operations, near-miss operations, and operation failures for each operational task were collected by integrating observers’ records and extracting videos. Task completion time was defined as the time taken by the participant to complete a task, measured from when the researcher finished reading the task instructions until the participant verbally reported the task completed. Frequency of manual query referred to the number of times a participant consulted the manual during the task due to encountering difficulties. The frequency of asking for assistance was the number of times a participant sought assistance from the researchers while completing the task. According to the National Medical Products Administration guideline [17], a difficult operation is referred to as task completion that meets requirements but falls short of expectations, such as prolonged operation times or reduced efficiency. This also included tasks completed by chance. Near-miss operation refers to tasks completed under conditions where unacceptable risks were nearly encountered. Operation failure referred to user tasks that did not meet the expectations or were not completed, including instances of exceeding time limits, making mistakes, omissions, or interruptions. Descriptive statistical analyses were conducted on the behavioral and subjective data. The content analysis method was used to analyze the interview data. All interviews were audio-recorded and transcribed verbatim to create a textual dataset for analysis. Furthermore, 2 researchers (YW and KL) independently reviewed transcripts for coding. Any discrepancies were resolved through discussion.

### Ethical Considerations

This study has been approved by the Ethics Committee of Tianjin University (TJUE-2024-278). All participants provided written informed consent. Each participant received 200 RMB (approximately US $28.64) as compensation for their time and contribution upon completion of the evaluation. All participant data were kept confidential and anonymized in accordance with the approved ethical protocol.

## Results

### Operational Task

Tables 4 and 5 present the task completion time and frequency of manual query, asking for assistance, difficult operations, near-miss operations, and operation failures for each operational task. Task 5 (priming the infusion line and setting up an infusion in drip mode) recorded the longest completion time (149.8 s) and was associated with considerable operational challenges. For example, nurses found it difficult to access the infusion mode function and encountered issues with priming the infusion line, which has extended task completion time. Similarly, task 1 (installing the pole clamp, portable handle, and fixing the pump to the stand) and task 6 (setting an infusion in time-dose mode) exhibited a high mean completion time and substantial operational issues. We observed that participants encountered difficulties in installing accessories, which extended the task completion time. Additionally, some participants spent excessive time when using the time-dose mode for infusion setup due to not understanding of the terminology presented on the screen. In contrast, simpler tasks, such as task 2 (installing the power supply and turning on the pump), were completed quickly (26.2 s) with minimal operational issues. Task 3 (installing the infusion line), task 4 (adjusting the brightness level), task 7 (setting an infusion in mL/h mode), task 9 (setting an infusion in safe mode), task 11 (turning off the pump and removing the infusion line from the pump), and task 12 (removing the pole clamp and portable handle from the pump) were completed in moderate times but still involved several operational issues. Alarm-related tasks also showed varied performance. Task 10 (addressing a power connection alarm) was completed relatively quickly with less operational issues than task 8 (addressing an air bubble alarm).

**Table 4:** Task completion time for each operational task.

Task	Time (s), mean (SD)	Time (s), median (IQR)
1	134.3 (70.3)	142.0 (62.3-192.3)
2	26.2 (5.9)	25.0 (21.5-29.0)
3	46.2 (20.4)	39.5 (33.5-54.5)
4	43.9 (44.8)	22.5 (19.3-51.0)
5	149.8 (130.5)	97.0 (61.5-190.8)
6	134.0 (91.8)	97.0 (57.3-234.8)
7	69.4 (58.7)	49.0 (32.3-73.3)
8	94.4 (36.1)	101.5 (52.8-119.0)
9	44.3 (16.9)	38.5 (30.3-58.5)
10	36.8 (32.0)	25.5 (14.5-42.0)
11	65.0 (14.2)	60.0 (54.8-80.5)
12	47.6 (38.2)	30.0 (22.8-51.0)

**Table 5:** Frequency of manual query, asking for assistance, difficult operations, near-miss operations, and operation failures for each operational task.

Task	User manual query, n	Asking for assistance, n	Difficult operations, n	Near-miss operations, n	Operation failures, n
1	5	4	1	0	13
2	0	0	0	0	1
3	1	0	2	0	0
4	3	0	14	0	0
5	4	1	16	4	9
6	7	0	3	0	9
7	2	0	8	3	1
8	4	0	8	2	1
9	1	0	7	0	1
10	4	0	5	0	0
11	0	0	11	0	1
12	4	0	4	0	0
Total	35	5	79	9	36

Of the total recorded operations (Table 6), difficult operations were observed most frequently (n=79), with alarm resolution (n=15), screen brightness adjustment (n=12), turning off the pump (n=11), and accessing the infusion mode setting function (n=11) constituting the most prominent challenges. Difficulties in starting (n=8) and pausing an infusion (n=7), along with number adjustment (n=5), were also notable. Near-miss operations, although less frequent (n=9), were primarily characterized by occlusion alarms due to the roller clamp not being opened (n=5) and the use of an incorrect infusion mode (n=2). A total of 36 operation failures were documented. A number of nurses were not able to install the portable handle (n=8) or the pole clamp (n=4). Critical procedural failures involved not priming the infusion line correctly (n=5), not starting the infusion after programming (n=5), and failing to notice or address system alarms (n=7).

**Table 6:** Type and frequency of difficult operations, near-misses, and failures.

Type		Frequency, n
Difficult operations (n=79)		
	Difficult to resolve alarm issues	15
	Difficult to adjust the brightness level	12
	Difficult to access to the function for infusion mode setting	11
	Difficult to turn off the pump	11
	Difficult to start the infusion	8
	Difficult to pause the infusion	7
	Difficult to adjust numbers	5
	Difficult to remove the portable handle from the pump	4
	Difficult to install the infusion line	2
	Difficult to access to the function for brightness level adjustment	2
	Difficult to install the pole clamp	1
	Difficult to program infusion under the time-dose mode	1
Near-miss operations (n=9)		
	Occlusion alarm triggered due to not opening the roller clamp	5
	Used wrong mode for infusion	2
	Air bubble alarm triggered due to incorrect priming	1
	Input wrong number when programming an infusion	1
Operation failures (n=36)		
	Installed the portable handle in wrong direction	8
	Did not notice the alarm or do not fix the alarm issues	7
	Did not prime the infusion line correctly	5
	Did not start the infusion after programming	5
	Unable to install the pole clamp	4
	Installed the power supply in a wrong way	2
	Input wrong number when programming an infusion	2
	Used wrong infusion mode	1
	Unable to install the portable handle	1
	Did not open the roller clamp	1

### Knowledge Task

Due to incomplete video recordings for 2 participants, the analysis of knowledge task was conducted for only 10 participants. All participants were able to complete the tasks within 2 minutes (Table 7). Among them, the accuracy of 4 knowledge tasks did not reach 100%.

**Table 7:** Completion time and accuracy rate of the knowledge tasks.

Task	Task completion time (s), mean (SD)	Accuracy rate (%)
1	60.2 (63.3)	100
2	82.4 (52.9)	50
3	98.3 (33.8)	60
4	59.3 (31.4)	70
5	91.6 (49.1)	100
6	35.1 (13.5)	100
7	75.7 (66.8)	90

### Subjective Perceptions

The mean scores for perceived ease of use, satisfaction, and use intention were 6.0 (SD 0.7) or above, indicating that participants perceived the pump easy to use, were satisfied with it, and were willing to use it (Table 8). The mean scores for perceived mental workload and perceived likelihood of making programming errors were 2.7 (SD 1.1) and 2.1 (SD 0.9), respectively, suggesting that the participants did not experience significant cognitive load while using the pump. The mean score for perceived concentration level required was 4.2 (SD 1.3), indicating that participants held a neutral stance regarding the need for high levels of concentration when using the infusion pump.

**Table 8:** Subjective perception results.

Dimension			Mean (SD)			
Perceived ease of use						
	Overall (items 1-6)	6.3 (0.5)				
	Priming and loading a set	6.0 (0.9)				
	Loading and unloading a set	6.3 (0.9)				
	Programming a basic infusion	6.7 (0.8)				
	Programming a drug infusion from scratch	6.8 (0.5)				
	Titrating ﬂow rates	6.2 (0.7)				
	Turning the pump on and oﬀ	6.0 (0.6)				
Perceived mental workload			2.7 (1.1)			
Perceived concentration level required			4.2 (1.3)			
Perceived likelihood of making programming errors			2.1 (0.9)			
Satisfaction			6.0 (0.7)			
Intention to use			6.0 (0.7)			

### Interview

#### Reasons for Difficult Operations, Near-Miss Operations, and Operation Failures

During the installation and disassembly phases, participants encountered difficult operations, near-misses, and failures in key tasks, such as pole clamp installation or removal, portable handle attachment, power supply connection, and infusion line loading. The primary contributing factors were the users’ unfamiliarity with accessory installation procedures and the absence of relevant instructional cues on the infusion pump or in its user manual (5/12, 41.7%). For example, as one participant noted,

I was unaware that the pole clamp required a pulling action. Even after consulting the diagrams in the instruction manual, I was unable to determine the correct method of operation.

P7

In system configuration, difficulties were observed in adjusting screen brightness and powering off the device. Several participants (5/12, 41.7%) reported that the brightness adjustment function was difficult to locate and there was no confirmation feedback after adjustment. For instance, one participant (P1) noted,

Following the brightness adjustment, selection of the ‘OK’ button provided no feedback, neither a return to the previous interface nor a confirmation, to indicate that the new setting had been implemented.

P1

The shutdown procedure also deviated from their familiar devices, resulting in prolonged completion times. For instance, one participant said,

Based on my prior experience with other infusion pumps, I expected that a long press would typically trigger a shutdown interface.

P11

During infusion setup, users experienced difficulties, near-misses, and failures in tasks including line priming, numeric input, mode selection, starting or pausing an infusion, and roller clamp operation. Several participants (4/12, 33.3%) indicated that the infusion mode setting function was not intuitively accessible, and terminologies were not clearly displayed, causing confusion. For example, one participant said,

I initially assumed that all mode selection options would be located within the menu system.

P12

Operational inconsistencies between this infusion pump and the participants’ routinely used devices further contributed to uncertainty in initiating and pausing infusions (1/12, 8.3%). P4 noted a key operational discrepancy:

With our conventional infusion pumps, the infusion typically starts immediately upon pressing the confirmation button. However, this specific model requires an additional step of pressing a separate ‘start’ key to initiate the process.

P4

Unclear button labeling also increased the cognitive load during programming (2/12, 16.7%). P11 expressed confusion regarding button functionality, noting:

I used the ‘OK’ button to confirm system status, followed by the ‘Pause’ button to suspend operation, though I frequently encountered difficulty differentiating between these two controls.

P11

Moreover, omissions, such as incomplete priming, not opening the rolling clamp, and incorrect value input, were observed (7/12, 58.3%). For instance, participants said,

Initially, I had located the mL/h mode, but subsequently realized that the drip mode was the required setting for this specific task.

P9

I recognized that the occlusion was caused by not opening the roller clamp.

P5

In alarm handling tasks, several nurses (4/12, 33.3%) failed to notice active alarms, while others who did recognize them often could not interpret the alarm messages or determine appropriate actions to resolve the issues. For example, one participant indicated:

I observed that it did not affect the infusion process, so I did not address it.

P5

P7 reported confusion regarding a system alarm, stating:

Initially, I did not understand the meaning of Preset Complete, and consequently could not comprehend the reason for the alarm activation.

P7

#### Suggestions for Improvement of the Pump

Participants generally reported a favorable overall impression of the pump, with most describing it as user-friendly, easy to learn, and featuring a clear interface (11/12, 91.7%). Representative comments included:

The overall interface is somewhat better than the infusion pumps I have used before; for instance, the numerical values are more prominent.

P1

It is quite good that I could start using it without much difficulty on the first try.

P5

It is mostly similar to other infusion pumps; I should be able to use it by exploring a bit myself.

P10

The Chinese language is easy to understand, and setting the modes is relatively straightforward.

P7

Regarding comfort and weight, participants found the pump generally comfortable to handle (10/12, 83.3%), with comments such as,

It is still quite comfortable, and the key sensitivity is good.

P1

Acceptable, and not very heavy.

P5

The keys are comfortable, easy to press.

P2

Regarding the button design, participants suggested improvements to increase layout spacing, reduce ambiguity, and clarify key functions (6/12, 50%). For example, participants noted,

The operation keys are too crowded; making them slightly larger or spacing them further apart might make operation easier.

P3

The alarm reset key are sometimes confused.

P4

The start/stop key and the rapid bolus key are too close; I initially mistook the bolus key for the start/stop key.

P5

The menu and back keys are confusing; I could not find the mode adjustment interface.

P6

Making the start/stop key more prominent would be better, as starting and stopping are our most frequent actions.

P9

The meaning of the menu key is not entirely clear.

P10

Additionally, participants identified issues related to confirmation feedback, accessories installation, and infusion set loading (5/12, 41.7%). P2 highlighted the lack of feedback during adjustments:

Brightness settings and numerical input require some confirmation feedback.

P2

P7 expressed concern over functional ambiguity:

The OK key and the alarm reset key are combined, which I find confusing; I prefer each key to have a dedicated function.

P7

Regarding accessories, P3 raised a safety concern:

In clinical practice, infusion pumps are often suspended. The ones we use are one-piece, so we are concerned about whether this clamp pull-ring is secure enough to avoid detachment.

P3

Participants noted:

The infusion lines on the pumps I used were loaded vertically; I feel gravity might work better that way.

P6

Vertical loading is more convenient; horizontal loading requires figuring out the direction, which takes extra time.

P8

Further suggestions for design enhancement included increasing alarm volume, improving accessory installation, and incorporating touchscreen capability (4/12, 33.3%).

The alarm volume seems somewhat low; it is not very salient.

P5

It would be better if the pole clamp was permanently fixed to the pump. In clinical settings, we rarely need to detach it, and removable parts can get lost.

P8

Clinical settings have many tangled lines; it would be more convenient if the power cord could be stored attached to the pump after disconnection.

P4

P11 proposed using a touchscreen:

A touchscreen would be preferable, making numerical input less cumbersome.

P11

#### Suggestions for Improvement of the Manual

Participants generally perceived the user manual as clear and detailed (10/12, 83.3%), while proposing specific enhancements to optimize its utility. First, 2 (16.7%) nurses suggested improving the readability by enlarging the font size and incorporating colorized interface diagrams to highlight critical information. For instance, participants mentioned,

The font size may be somewhat small for senior clinicians.

P1

The section illustrating the pump interface could be presented in color, matching the actual operational display, to facilitate easier use.

P4

Second, 1 (8.3%) participant advised consolidating alarm-related content by integrating alarm definitions with corresponding troubleshooting procedures. As P4 explained,

Placing the alarm descriptions and resolution methods on the same page would better support clinical workflow, since I typically identify the alarm type and immediately seek the solution.

P4

Third, nurses proposed adding a streamlined flowchart at the beginning of the manual to outline key clinical procedures (3/12, 25%). One participant suggested

…*extracting a 2–3 page quick-reference guide covering essential operations and alarm handling for direct clinical guidance.* [P3]

P8 emphasized the need for “*an initial overview with a simple flowchart before detailed textual explanations*.” Finally, 1 (8.3%) participant recommended supplementing certain operational details currently absent from the manual. P10 explicitly noted the absence of guidance on “how to install the pole clamp” as an example of missing critical information.

### Remaining Usability Issues and Potential Risks

Table 9 presents the summary of remaining usability issues, potential risks, severity level, and corresponding design recommendations.

**Table 9:** Remaining usability issues, potential risks, and corresponding design recommendations.

Usability issues	Potential risk	Severity level	Design recommendations
Did not notice the alarm; difficult and unable to resolve alarm issues	Delay in responding to critical alarms	High	Clearly display the cause of the alarm and the steps to resolve it
Used wrong infusion mode	Dosage error	High	Distinctiveness of modes should be improved
Input wrong number when programming an infusion	Dosage error	High	A second confirmation for parameters is required
Difficult to program infusion under the time-dose mode	Inefficiency in programming and potential dosage error	High	Implement step-by-step instruction and clearly explain the meaning of the terminologies on the screen
Did not prime the infusion line correctly	Potential residual air in the infusion line	High	Visibility of pump status need to be clearly displayed; provide clearly instruction for priming
Did not open the roller clamp	Treatment delay	High	Visibility of pump status need to be clearly displayed; remind users to check the openness of the roller clamp before starting an infusion
Did not start the infusion after programming	Treatment delay	High	Visibility of pump status need to be clearly displayed; remind users to start infusion after programming, if not started within a set period, a reminder alarm should be triggered
Difficult to adjust numbers	Inefficiency in programming	High	Clearly indicate the method for rapid value entry
Difficult to start and pause the infusion	Treatment delay or unable to quickly stop the infusion	High	Provide a distinct start and stop key with clear labeling
Difficult to install the infusion line	Treatment delay	High	Provide clear instructions and incorporate error-proof design features
Difficult to find the function for infusion mode setting	Potential infusion in the wrong mode	High	Clear navigation
Difficult to find the function for brightness level adjustment and difficult to adjust the brightness level	Misreading or incorrect operation due to unclear screen display	Middle	Clear navigation; provide confirmation and feedback after settings
Difficult to turn off the pump	Inefficiency	Low	Provide clear instructions
Difficult or unable to install or remove the portable handle or pole clamp or power supply	Unstable or inconvenient device movement; Unstable installation or device falling; Power failure	Low	Provide clear instructions

## Discussion

### Overview

This study reported on a summative usability testing process for an infusion pump. The overall usability level of the pump was assessed by observing nurses’ operating behaviors when using the pump. The study identified difficult operations, near-misses, and failures encountered during tasks, analyzed the causes of these issues through participant interviews, highlighted the design advantages of the infusion pump as well as existing interface design flaws, clarified usage risks, and proposed design improvement suggestions. These findings can provide valuable insights for optimizing the infusion pump design, helping practitioners define use risks and training priorities, and offering important implications for the usability evaluation schemes of medical devices.

### Main Findings

Generally, the examined infusion pump effectively assisted participants in completing the tasks. Participants were able to complete all operational tasks within 3 minutes, indicating high overall operational efficiency. For knowledge tasks, although all were completed within 2 minutes, suboptimal accuracy was observed in a portion of tasks. In most cases, the participants completed tasks independently. They generally found the infusion pump to be user-friendly, requiring a medium level of attention resources, and reported low levels of mental workload and likelihood of making programming errors.

However, participants encountered several difficulties when operating the infusion pump, reflecting that there were still some design deficiencies that needed to be addressed. First, the infusion pump should increase the visibility of system status [28]. This issue has also been reported in other studies on infusion pump usability [21,23]. Low visibility of system status may prevent nurses from accurately determining the operational status of the infusion pump, thereby potentially leading to adverse events. Second, function navigation should be improved. Some nurses had difficulty in locating brightness adjustment and infusion mode setting functions. Liu et al [21] also observed that certain functional menus of infusion pumps were difficult to access. This may be attributed to a misalignment between the system interaction flow and user expectancy. A clear and well-structured navigation system can prevent user errors during operation, while excessive navigation options, overly deep menu hierarchies, and ambiguous categorizations can significantly increase users’ memory load. Third, the infusion pump interface should be aligned with the mental model of target users, and the operating logic of different infusion pumps should be as consistent as possible. Some nurses reported differences in operational logic compared with pumps they regularly used, resulting in increased difficulty and errors during usage. This constitutes a violation of the consistency principle in human factors design [29]. When nurses encounter an interface that does not align with their established mental models, they are compelled to relearn and reconstruct new cognitive schemas. Fourth, many participants neglected alarms or were unclear about their meanings, which poses significant risks in clinical use. Previous research has indicated that a significant number of nurses failed to comprehend the meaning of infusion pump alarms clearly, leading to untimely or absent responses [21]. This represents a violation of the human factors design principle that systems should support users in recognizing, diagnosing, and recovering from errors. Effective error messages should use plain, unambiguous language, precisely identify the nature of the problem, and offer constructive, actionable guidance toward its resolution. Alarm design can also consider combining visual and auditory signals and avoid masking effects to ensure quick perception in complex clinical environments. Fifth, a few participants made errors in entering infusion parameters or using incorrect infusion modes. Other researchers have reported that confusion regarding dosing units and/or concentrations when using infusion pumps can lead to medication overdoses [22,30]. Input errors pose serious risks to patient safety, and while some infusion pumps include features to detect unsafe infusion rates [31], such errors may not be entirely avoidable through system design [5]. Finally, the clarity and comprehensiveness of the manual should be improved. Participants noted that a combination of images and text might enhance understanding more than using text alone.

### Implications

Our study results provide several important implications. For clinicians and educators, the findings pinpoint areas requiring focused training and competency assessment, moving beyond general device familiarity to targeted practice on high-risk, error-prone steps. The problem analysis provided concrete criteria to inform procurement decisions, allowing buyers to compare devices based on specific safety-related usability attributes rather than features alone. For manufacturers, the results offered actionable design improvement recommendations directly linked to mitigating identified use errors, supporting a user-centered design and risk management process as mandated by regulatory frameworks.

### Limitations

First, due to constraints related to the duration of the experiment and the availability of participants, we only recruited 12 participants. Given that our primary objective was to identify predominant usability challenges rather than to quantify rare events, this sample size was considered sufficient to capture the majority of issues. Future studies can consider increasing the sample size to ensure statistical power and generalizability. Second, no alternative system interfaces were assessed; therefore, no comparable results were available in our study. This may limit our findings and ideas on system interface improvement. Third, the sequence of tasks was designed to reflect the standard clinical workflow for operating an infusion pump; however, it may induce potential order effects. Future studies can consider randomizing the order of the tasks. Fourth, the impact of training on infusion pump use was not considered. Fifth, the experiment was conducted in a quiet simulated ICU, and nurses’ use performance in real clinical scenarios (eg, noise, interruptions, and multitasking) may differ from the testing conditions. Finally, future tests could integrate devices like eye trackers to study the impact of infusion pump interface design on users’ visual attention allocation.

### Conclusion

This study recruited 12 nurses with infusion pump experience to conduct a simulated usability test of an infusion pump. It not only assessed the current usability of the infusion pump but also identified the root causes of user errors by combining subjective and objective data. This approach can help mitigate potential interaction design issues that could lead to user errors, providing important implications for the design and evaluation of medical devices.

## Data Availability

Data are available on request from the corresponding author.

## Abbreviations

ANSI: American National Standards Institute
AAMI: Association for the Advancement of Medical Instrumentation
FDA: US Food and Drug Administration
ICU: intensive care unit
IEC: International Electrotechnical Commission
